# An Autobiographical Sketch of a Drunkard

**Published:** 1858-07-01

**Authors:** 


### Art. V.—AN AUTOBIOGRAPHICAL SKETCH OF A DRUNKARD.

With truth I may call myself a drunkard from my mother's womb. On November 29th, 1803, after a long and severe labour on the part of my mother, I was brought into existence, some twenty minutes previous to which, my mother, taking advantage of the absence of her accoucheur, got out of bed, and, impelled by an uncontrollable desire, took from the cupboard a bottle of brandy, of the contents of which she swallowed a large wineglass.* Now, this act, at such a time, for a woman fond of or addicted to drinking, would not have been considered extraordinary; but for one of her well-known abstemious habits, who had, probably, never before tasted undiluted spirits, could only be accounted for from that morbid and depraved taste which sometimes prevails with the sex on like occasions. However, such act was the prelude to my after-existence. Now, whether the same cause that produces diseases such as gout, stone, consumption, and various other maladies to which suffering humanity is liable, traceable in descendants to their parents, acted so on me in producing that predilection for alcoholic drinks or not, I will leave for the physiologist and curious in such matters to determine.

I will now begin briefly to relate the chequered events that have befallen the poor inebriate, up to the time that the grace of God changed his career, I hope permanently, by means of the truth promulgated through the doctrines of teetotalism, together with the mysterious revelations of the punishment awaiting the drunkard in the kingdom of darkness, when he goes hence.

Soon after my birth, as my mother's health did not permit of her nursing me, I was given to a person living some eight miles from the place of my nativity, whose house was situated in the midst of a forest of exquisite rural and sylvan beauty. Many years since have I gone to review its romantic scenery, and endeavour to realize the ideas and feelings and reminiscences of my early days;† brought to remembrance the gipsies' camp—the shrinking, half afraid, from the extended hand of the old crone, as she took the jug of milk, and kissed the averted cheek of the little curly-headed child, held in the arms of the kind-natured foster-parent, who always supplied this luxury to these

* The communication of this fact I heard made to a female friend by my mother, whilst contemplating me as I lay in a half-drunken, half-sleeping state on some chairs.

† Those in age, who recollect the tenacity of their very early childhood impressions, will not discredit this assertion.

people, when periodically encamped under their old favourite beech-tree, which they always selected under which to rendezvous at certain seasons of the year. At such times, many a maiden coy and bashful swain were wont, at evening's close, by crossing with silver the sybil mother's palm, to elicit from her the coming destiny of weal or woe that lay in wait on their further path through life. Driven by the necessity their rambling life enjoined, one bright trait redeemed the deception of half its sin—the magic skein unravelled, no future fate foretold, that brought to the maiden's cheek the blush of shame, or dimmed with tear of sorrow her sparkling eye; half doubting, yet hoping that the tale was true, a laughing pleasure beamed from her joyous face, that partly realized the anticipated bliss. Ah! happy days of inexperienced boyhood—no more amid the stern realities of life shall such like scenes return! How, alas! they contrast with the years of sin and folly, and followers in their train—sorrow and remorse—that since have beset my path; and I cannot but here repeat the prayer that spontaneously burst forth, on the first conviction of sin, in early days:—"O, Virtue! would I had ne'er swerved from thee, thou goddess whose bright smiles do cheer the weary sojourner on earth, making his rough way smooth. But now, thy rival, Vice, clinging as serpent's coil, in soft and slippery folds, by his close grasp unhinging all the nobler faculties; and the seeds, O, Virtue! thou didst sow, are, in embryo, just bursting, crushed by the pressure."

But to resume my narrative. After remaining at my dear old nurse's till about six years old, I recollect, one evening of a beautiful day of summer, a post-chaise being driven up to the wicket-gate of the little farm, and a lady getting out that I had been told to call mother, but whom I had so seldom seen that I could hardly reconcile to myself that it was right to call her by that endearing name, in the stead of my dear old nurse, who had shown all a mother's love to me. I was immediately got ready, and in the midst of bewailings and tears, left my foster-parents' roof, no longer to be a home to me. There was one circumstance in her—my nurse's—habits of life, which here I must not omit to mention, from its singularity—in contradistinction to my after-habits of life—and that was, she never drank any beverage but tea or water. No power on earth could make her partake of any alcoholic drink; she did not like it; she had a natural aversion to anything of the sort from her infancy. She took no merit to herself for her abstinence; and "total abstinence," as a moral virtue and enjoined practice, was then unknown. Her husband, a small farmer, on the contrary, often came staggering home in his cups from market, and she, woman-like, did not fail to well scold him the next morning for his irregularity.

In a few days, under the caresses and kind treatment of my parents, I became perfectly reconciled to my new home; I no longer sorrowed for the absent. There was one little novelty, I recollect, which tended much to reconcile me to the change, and that was, the fine trappings my father occasionally wore as sergeant in the Yeomanry Cavalry, at that time in constant requisition, apprehending an invasion by Bonaparte—a name fraught with terror to my young ears. Many curious tales of those days, in after-years I recollect being told, about certain individuals of the troop showing the "white feather" on a certain night, when called to horse with the formidable announcement of the French fleet being in sight—information derived from the lighting of the agreed signal-fires on the neighbouring heights. The few large vessels in the Channel which gave rise to all this commotion, proved, at last, to have been only some of our "merchantmen." One gallant soldier was suddenly seized with the gout; a second had scalded his leg; a third, his horse was suddenly taken lame;—for which excuses, I need hardly say, they had to bear the ridicule of their townsmen for some years after. But to proceed from this digression. In due time I was sent to school; and after five or six years of plain education, learning land-measuring and other essentials, to fit me for what I was then intended—a farmer—I left school a hopeful lad, possessing the love and good wishes of my master, and all who were acquainted with me. There was, I recollect, in those early days, one trait in my character which, from what I have hereafter to relate, will not be considered in me vanity now to mention, and that was, a love of truth. I was never known to tell a wilful lie. My parents were not, strictly speaking, religious people. I was sent to my church, taught the catechism, and, at the proper time, confirmed; but the sin of telling a lie had been so particularly impressed on my mind, that that sin I was rarely guilty of; and even to this day, that early impression, in the midst of all the complicated sins which follow in the wake of the drunkard's career, has been respected, and the sin held in abhorrence from that early conviction. From this I have often thought how necessary for parents and guardians of youth, or rather children—particularly as the mind at that early age is fitted for receiving and retaining impressions of what is good and evil—how urgently should be instilled in their young minds a hatred of all sin, as an offence against a pure and holy God. If such practice more generally prevailed in the teaching of little ones, I am satisfied, from my own experience, howsoever in the heyday of youth and folly such instructions may be forgotten, that the time will arrive in God's providence when the seed so sown shall revive, and bring forth fruit unto repentance.

On leaving school, I commenced my duties on the farm, during which, manhood began to dawn upon me. At this time, one particular occupation, at which I was occasionally engaged, tended to increase my love for "that accursed thing which worketh abomination,"—"that vial of wrath," mentioned in the Book of Revelations, "that should be poured out upon the earth,"—"that produce of the vine of Sodom and Gomorrah,"—"that poison of dragons and the evil venom of asps," which biteth like an adder and stingeth like a serpent,—"that abomination that maketh desolate." This occupation was the driving of post-horses; for, in addition to my father's business of a farmer, he united that of a postmaster, and frequently, when the regular drivers were out, I was found very useful in taking a pair of the remaining horses, and executing any order that might come in. This accelerated my progress to becoming a confirmed lover of strong drink. Being naturally good-natured, and not wishing to show on such occasions that I felt myself above the menial duties I was employed in, I was led, unhesitatingly, to accept the contents of the proffered glass. From this data, and from this cause, I reckon my gradual progress to ruin. This alternate employment continued till my twenty-second year, when my father took possession of an additional farm, a few miles from the town in which he lived, intending, after realizing a partial return of the capital used in stocking the farm, to give it into my hands. In the meantime I was, under his jurisdiction, to manage the business, my remuneration for which was to be the produce of a dairy of eight cows, piggery, poultry, and other advantages, together with the farm-house to reside in, free of rent. I had not been thus settled more than a year, when I formed an attachment for the daughter of a farmer retired from business, whom, after years of wooing, I married, looking forward at that time to many years of peace and happiness from the affection of an amiable and beloved wife, whose excellent disposition and domestic acquirements were well qualified to give a home that charm which, to one of regular habits and well-formed mind, could not fail to be appreciated.

I mentioned before, that I had always been brought up in a belief in the doctrines inculcated by the "Episcopalian Church of England," and revered her formularies,—sitting under a minister of good moral habits, but, in his preaching, advancing very little of that evangelical *life-saving truth* which, I thank God, now more commonly prevails with the ministers of the "Establishment." In those days, when the truth as it is in Jesus was preached in all its faithfulness by a Church of England minister, he was branded, forsooth, as a "Methodist,"—intended as a term of reproach,—and many of his congregation, particularly amongst the higher circles, would, on his ascending the pulpit after the "prayers," abruptly leave the sacred edifice. Of this I had ocular demonstration, shown to the person of a reverend gentleman who, in consequence of this treament, together with a slight change of views, seceded from the Church, and afterwards became a popular Baptist minister, dispensing the bread of life to a large congregation in the metropolis, and but very recently deceased.*

About this time I accidentally heard a young man, an intended missionary for Persia, who had previously been carrying on business as a chemist, the power of whose preaching was such, that by God's blessing a great impression was made on my own and wife's mind;—on hers, thanks be to God, a permanent one; mine, the sequel of my history will show. After this, my old church minister seemed to have no life in him. I in vain listened for those quickening truths, the faithful preaching of which makes the Saviour all in all to the poor sinner, and by whom *alone* cometh salvation; but, alas! too much dependence on creature righteousness was preached, and too little seeking for God's grace, by the gift of his Holy Spirit, to will in you to work and to do his good pleasure, was his characteristic. The impression made at this time has never been entirely obliterated, notwithstanding the assaults of the "wicked one" have triumphed for a time in bringing me under subjection to his will. Thanks be to God, he has always in his mercy made my sins my reprovers, by using them in the last extremity to bring me to a conviction of the heinousness of their nature, in contradistinction to the emanations from a pure and holy God, whose pervading influence constrains the sinner to seek pardon by pleading a Saviour's atoning blood, the imputation of his righteousness, the sanctification of the third person in the Trinity, the Holy Ghost, imparting to me his spirit to love holiness and hate sin.† In looking back at the serious convictions of my past life, evanescent though they were, I can clearly see that God's grace was never withdrawn from me, nor will be from any convinced sinner, till he ceases to employ the means that God has provided for the preserving of that gift,—the ceasing to pray, and the keeping from attending that place in which he has said, "where two or three are gathered together, that he will be in the midst of them."

At this time, having nothing more to do with driving, I had, humanly speaking, a good opportunity to divest myself of that

* The late Rev. T. Evans, minister of John-street Chapel, Bedford-row, and succeeded in the ministry by the Rev. Baptist Noel. Many years since, the Rev. Mr. Evans was officiating clergyman at Milford, near Lymington, Hants.

† It must be understood that these were the doctrinal points in which I believed, and had convictions, at the time these incidents occurred. I by no means identify myself with the whole of them at the present day.—Sept. 3, 1853.

baneful habit, indulgence in strong drink, and did make many strong efforts for that purpose. But such resolutions, being based on my own creature strength, lasted but a short time; the temptation that beset me at the evening meeting, after the farmers' weekly market in the neighbouring town, proved too great for my weak resolves, and after a short interregnum of sobriety, I seldom arrived home from them perfectly sober. This went on amid repeated resolutions of amendment and relapses, till the third year of my occupancy of the farm, when, from the great and sudden depreciation in the value of agricultural produce, farmers of small capital were, of course, injuriously affected—their rent, on lease, having been fixed on an average of prices when corn realized double the amount, brought many a farmer to ruin, or to emigration, with the fragment of his property that remained. This so far affected my father's circumstances, as, instead of giving me possession of the farm, as he had previously intended, obliged him to throw up the remainder of his unexpired term into the hands of his landlord; so that no resource now seemed left to me for the maintenance of my wife and increasing family, but a return in some way to that sort of employment into which I had formerly been initiated,—to which no insurmountable difficulty presented itself, from my father still continuing the posting business, enabling him, by horsing a not very profitable coach, to procure for me the situation of a stage coachman on *another coach*, on a more profitable line of road,—the losses that he sustained by horsing one being considered as a sort of premium for the lucrative situation that I held on the other. This took place in 1831-2, and I now had a prospect before me, with moderate carefulness, of realizing a very comfortable living, and of providing for the exigencies of the future—my situation realizing four and five pounds per week. This post I continued to fill without accident for two years, when an event took place which was the indirect means of bringing me to a stand-still—that was, the death of my father; the direct cause of which, although the relation will form a short episode in my narrative, may not be thought out of place here to mention. The before-mentioned causes, which produced the necessitous circumstances which drove my father to leave the farm—a business to which he was devotedly attached—produced, from continually brooding on his loss, a weakness of mind bordering on aberration of intellect, from which he was just recovering when an election of a member of Parliament, for the borough he resided in, took place,—when, the usual dinner on such occasions being given, he, with the other *immaculate, independent voters*, was invited, that indulgence in their gourmand propensities and powers of imbibition, might render less keen their talents for entering the political arena, for the purpose of discussing the principles of him whom they were about to invest with the authority of their representative.

He came home from the *feast* in good time, his usual moderate habits and consideration of his recent illness, no doubt, preventing him from undue indulgence, and from the too common excesses committed on like occasions. Would that the same caution had been observed on the second day, when the party met for the purpose of *lunch*, or rather for that of consuming the fragments that remained. The very little that my father had drunk the preceding day being more than on ordinary occasions, produced—as the *use* of intoxicating drinks always does—that depression of the feelings which nothing but a return to the cause of the evil seems to give a transitory relief; the consequence was, his being brought home late at night by a good-natured, half-reeling friend, and laid powerless on the floor of the parlour. This ultimately produced paralysis in certain bodily functions, and in eight days from this—the day of enjoyment—death released him from his sufferings, of which event I was the next morning apprised, by a person sent to relieve me from my duties on the coach I was driving, so that I might be enabled to hasten to my mother and family, to join and sympathize with them in their affliction.

After the funeral, my late father's affairs were investigated, and found to be in that state which left hardly sufficient, after his creditors were satisfied, to enable my mother to carry on the posting business for the maintenance of herself and the junior branches of the family. This induced (my late father having been an old and respected inhabitant of the town for upwards of thirty years) the influential inhabitants of the neighbourhood to raise a subscription in her behalf, for the purpose of relieving her temporary difficulties, and more permanently fixing her in the business in which she had heretofore been engaged. This having been accomplished, matters proceeded in their usual course until the executors of my late father, finding that the coach before-mentioned, which had continued to be horsed for my benefit, was such a losing concern, that they were compelled, in justice to the junior branches of the family, to give notice of its discontinuance. The consequence was, the proprietor having no longer self-interested motives to induce him to continue me in his employ, very shortly afterwards took advantage (which I am sorry to say that I too frequently gave him) of my occasional fits of intemperance to remove me from my situation. I should have soon been reduced to great extremity, but for the kind individuals who had already so materially assisted my mother; they thought me severely treated, and got up a further subscription towards establishing a coach, that should leave the town at a later and more convenient hour, as a feeder or branch of that coach which left a large town eighteen miles distance, for the metropolis, at about mid-day, to which situation I was duly appointed.

This act gave such offence to my late employer and to the body of proprietors, that they used every means in their power to render the undertaking unsuccessful. This coach continued to run for upwards of twelve months, when, notwithstanding the exertions of my well-wishers and friends, the pecuniary loss was so great that it was ultimately given up. I was then under the necessity, by way of keeping the wolf from the door, of officiating as driver for my mother, whose scanty wages—her business not affording her the means of giving me more than an indifferent person—were barely sufficient even for that purpose. This derogatory employment, fly and post-chaise driving, continued but a few months; when, from the interest employed by some unknown friend, I unexpectedly received a letter from a large coach proprietor, appointing a meeting on the succeeding day at a town about twenty miles distance, which, with most sanguine anticipations, I most readily kept. On seeing him, he told me "that through the interest of some influential party, he was induced to make me an offer of driving a coach from S——n to W——th, a distance of seventy-five miles per diem, down one day and back the next, in the room of an old part-proprietor and servant, whose ill-health obliged him to leave, with but little hope of ever again being enabled to resume his duties." He added, "Now, take care what you are about; be steady; you have as good a place given you as on any line of road that I am master of. I have heard that you are given to drinking; I hope that you will show by your conduct that the accusation is false." I warmly thanked him, and on the succeeding Monday took possession of my "box." I was now completely in my element; the coaches I had driven before were over comparatively short distances, and through monotonous scenery, but now I had seventy-five miles a day, embracing as great a variety of picturesque scenery as the eye would wish to rest on. I removed my wife and family to W——th, one of the towns of my destination on alternate days, at which I spent the Sunday. This town was situated in a beautiful bay, like what I have heard described of the Bay of Naples, wanting the high ground of Vesuvius in the distance. My wife and little ones, from low spirits, impaired health, and countenances of sickly hue, shortly obtained that buoyancy of spirit, and that ruddy complexion of countenance, which proximity to the seaside so frequently produces.

There I might have been seen, on a Sunday afternoon, walking with my wife and children on the beautiful esplanade formed on the beach, envying not one amongst the many of rank and fashion that resorted to that delightful spot to inhale the renovating breeze from off the English Channel. I wonder not at good old George III. selecting this spot for his summer bathing residence. Its somewhat secluded and unostentatious beauty with much advantage contrasted with the glaring display of the rich mansions and bolder scenery of B——n, and the more fashionable watering-places of the present day.

This pleasing daily employment—leaving at about a quarter before eight in the morning, and reaching the termination of my journey before five o'clock in the evening—continued about fifteen months, with nothing to mar the health-inspiring exercise but the frequent excessive use of that accursed thing to which I have before alluded. It seemed ordained to be my curse; it tended to neutralize all the pleasing qualities of my better nature. The passing exclamation, when my name was mentioned in connexion with this vice, was, "What a pity!" In my past early experience of mankind I cannot, like many of the disappointed ones on life's stage, exclaim on the uncharitableness and undeserving fault-finding propensities of my fellow-man; on the contrary, I received at that period of my life the good wishes, sympathies, and much assistance of a substantial nature, from those on whom I possessed no claim. I found many more ready to overlook and make excuses for my failings, which I by no means deserved, than to reproach me according to my deserts. At the expiration of these fifteen months, the former driver of the coach, who, as I have before mentioned, was also a part-proprietor, contrary to all expectation, recovered sufficiently to enable him, by a great effort, again to resume his "seat;" and by keeping a helpmate by his side to afford him occasional relief, managed to maintain his situation. This again obliged me to turn my attention to other means of procuring a livelihood for myself and family. After six months of idleness, returning from an unsuccessful application for the management of a charity Foundation School, for which a master was advertised, and for which, from my previous habits and employment, I was by no means fitted, I accidentally heard of a gentleman, a magistrate, wanting a coachman. On the first intimation of this, the feelings of pride were aroused in my "Adamite nature," which were somewhat lessened on my being informed that "livery" was not required to be worn. A house on his estate was provided to live in; and fortunately no waiting at table or other more menial duties were required, for which art and mystery my previous avocations in life did not at all befit me. Driven by a necessity which admitted of no choice, I, with a hesitating step and awkward mien, solicited of this gentleman the favour of filling his vacant situation. As he had previously known me when driver of the coach, he commiserated the extremity of my position, which enforced my seeking so menial an employment, and expressed his fear that the contrast of his service with my former one would be too great to give mutual satisfaction. I succeeded in removing his scruples, and was forthwith engaged at a liberal wage, and additional perquisites of milk and vegetables. This, to a man of careful and provident habits, would have sufficed to render his life endurable till better things turned up; but I am sorry to admit that my old expensive and wasteful habits still so far influenced me, that some time was necessary ere I was disciplined into compliance with more frugal fare.

But, thanks be to God for His mercy and goodness in adapting one's mind and feelings to those changes in our temporal condition in life in which it is His pleasure to place us. My home was now situated at the northern extremity of a large park, about half a mile from the mansion; and many times have I looked with pleasure on my little ones lightly tripping across the park, on a fine summer's morning, to the dairy, for a supply of milk for their breakfast, and the day's consumption. The house, though small and inconvenient in some respects, with its neat flower-garden, and trellised lattice frame-work that covered the walls outside, over which crept the woodbine and sweet-smelling, white-flowering clematis, gave a humble charm and beauty to its appearance, which tended in some measure to reconcile us to its in-door inconvenience. There was one incident occurred during the time I was living with this gentleman which, as a faithful chronicler of actual events, degradatory though they may be, I am bound to mention. He kept two gardeners—the head one, a tall, bony Scotchman, of hard physiognomy and cold repulsive manners, who resided in the park at an adjoining lodge to my own. Whether the kind treatment my wife and children received from the family at the house, or from what other cause, I know not; but this man conceived an aversion to me and my family, which his bearing and manners too evidently showed. However, as long as his dislike was passively confined within his own breast, I cared but little for the ill-feeling. But time, at last, as in all similar cases, seldom fails to develop the venom, where such disposition is encouraged. It so acted on him as to produce the following *diablerie*. I had heard several complaints coming from this man of repeated losses of different vegetables from the kitchen-garden, particularly of the loss of one or two dozen of the sort of cabbages used for pickling. As it was no business of mine, I took but little notice of the matter till my attention was called to an evident alteration of manner in Mr. S., my employer, towards me, and an inquiry made of me by a gentleman, a relative, residing with him, of "whether the shoes that I wore in stable were nailed?" to which I answered in the affirmative, wondering at the drift of such question. This difference of behaviour on the part of my employer having lasted some few days, I had resolved to ask him if I had unwittingly given him any offence, when I received a message from a young neighbouring farmer, with whom I had been schoolfellow, to wait on him. So doing, he apprised me that suspicion existed in Mr. S.'s mind relative to my having something to do with the various robberies that had been effected from his garden: to use his own words, he said—"When I heard Mr. S. suspected you, I was determined, at the risk of being myself implicated for receiving things knowing them to be stolen, to state all I knew of the matter. The gardener came to my house, and, on seeing me, pulled from under his coat four large red cabbages, which he presented me, at the same time asking me if I would lend him a large hamper to pack up some crockery, which I accordingly did. Two days after this, who should I see but this same man and his son, bearing between them the identical hamper which I had lent him, filled with what appeared, from a leaf protruding from under the cover, to be 'pickling cabbages.' This took place at the little inn in the village, where the branch London coach changed horses (and which coach, by-the-bye, I was afterwards destined to drive). The hamper, with its contents, he immediately put on the coach, and booked it to some person in London. Now," he added, "you can mention this circumstance to Mr. S. at once, or wait till you are directly accused with the crime." I resolved to adopt the former method, for which purpose I sought Mr. S., apologized for the interruption, told him I had suffered much uneasiness from the alteration in his manner towards me, which I attributed to some person endeavouring to disparage me in his estimation. He then told me that "his gardener had informed him that on frequent occasions he had missed vegetables from the garden, and that the week previous two dozen of cabbages had been stolen. On my asking the man if he had any suspicion of any one, he told me, 'that comparing your stable shoes with the foot-marks on the ground, that the impressions of the nails agreed exactly.' Still (he added), Mrs. S. and the family will not believe it, nor should I have entertained the suspicion for one moment; but I am sorry to have noticed lately a sort of looseness of manner in you, which I had never noticed before, and which I am assured is produced from a too great indulgence in strong drink; but, however, for the future avoid that evil." Thus did this gentleman humanely and condescendingly speak to me of my faults. On hearing the information I had to give, he said, "I know Mr. E., from whom you received this information; I will see him, and take the proper steps to bring the guilty party to justice." I was about to leave him, when he suddenly added, "He likewise accuses you of stealing bundles of wood from the 'pile,' on your way home through the park in the evening; but say nothing of this interview with me, and I will find means of eliciting the truth." Accordingly, by setting a person to conceal himself near the "wood pile," he found that this gardener and his son were in the constant practice of taking home, nightly, two faggots of wood. Mr. S., though a magistrate, and sworn to administer justice and repress crime, from humane considerations of the distress and misery that would be brought on this man's young family by a prosecution, satisfied justice by simply discharging him from his service. Thus was this man punished, and I hope, ere this, brought to repentance, and led to see that even in this world the wonder-working hand of God's providence makes our sins to find us out and prove our correctors.

After the above circumstance had passed over, I went on very comfortably in the quiet duties of my position, retaining the confidence of my employer, until my frequent indulgence in my old propensity placed him under the necessity of reluctantly giving me the usual notice to leave his employment. Business calling him to the town in which my mother still continued to carry on business, he informed her of his decision, remarking at the same time, that my love for stimulating drinks was such, that he thought a certainty of death itself would not wean me from the habit. This was in the year 1834, within about a month of my wife's confinement with her fifth child, in consequence of the proximity of which I was suffered to retain my situation till she had gained sufficient strength to undertake the fatigue of removal.

It was during one of my outbreaks whilst living with this gentleman that I first felt the symptoms of "delirium tremens," or rather, a sort of "magnetic clairvoyance;" it was not so severe in its nature as the former malady. The feeling developed on this occasion I shall be somewhat minute in describing, as it forms one of a series of evidences in what I have hereafter to relate of its producing that clairvoyant power on the soul or mind which realizes a telegraphic communication of the "inner man" with the immaterial essences of the invisible, or spiritual, world. On returning one evening across the park to my home (not inebriated), I was suddenly surprised by hearing the voices of a number of persons behind me, upbraiding me with my conduct, not only on account of its sinfulness in the sight of God, but as calculated to bring misery and suffering on my innocent wife and children. I seemed to hear the utterance of every word clearly and distinctly. The different bacchanalian orgies of my past life were narrated from one to the other with the greatest exactness. I stopped for these supposed railers to come up with me; but no—they approached no nearer—they seemed to stop likewise. Having a friend living in an opposite direction, I turned with the intention of visiting him, hoping by so doing to get rid of the annoyance; but in vain, for still they seemed to follow me. On crossing a brook, the gurgling ripple of its waters over the pebbly bottom beneath, formed words and sentences of reproach to confound me. The breathing zephyr, as it slightly rustled amongst the foliage of the trees, joined its voice to the streamlet below in my condemnation. All this, at last, satisfied me that it was no mortal whispering that I heard; and under this persuasion, without visiting my friend, I returned back to my home, determined, from the fear of ridicule, to keep my mental hallucinations, as they no doubt would be called, within my own breast. In this resolve I succeeded, and in the course of a day or two my mind recovered its usual tone. Within a week of the expiration of the time for me to leave this situation, I was informed by the landlord of an hotel of an adjacent town, that a gentleman in the neighbourhood, having bought a pair of young horses, wanted a person to break them into double harness fit for his own driving. I made application, and succeeded to my wishes, recognising in him a gentleman that I had ridden with side by side after many a good fox in years gone by—a sport that my indulgent father, fond of himself, allowed me occasionally to partake of in the days of his prosperity. I gave my employer such satisfaction, that he continued to keep me with him long after completing the object of my engagement; and I should have most probably remained with him, I know not how long, had not my desires led me continually to importune, by letter, the coach proprietor I had formerly served, and who, on the return of the invalid coachman before-named, had promised me the first situation he had it in his power to bestow, which promise he at last fulfilled, chiefly owing to the kind interposition of the very gentleman I was at the time serving. I look back, even now, with a feeling of pleasure to the six or seven months' services I rendered this gentleman, having left him, much to my surprise, without any one dereliction of duty calculated to neutralize the good feelings or alter the good opinion he had entertained of me.

The Autobiography of a Drunkard contains in it the most monotonous, uninteresting detail of events that can be possibly imagined. The repeated indulgence in his degrading passion rapidly produces that obtusity of intellect under which the vivacity of mind and the nobler aspirations of his better part—the soul—become so stultified as to prove a barrier to the full development of the nobler faculties of his being. Thus it was with me; I loved all mankind, and never did the cry of distress reach my ears, or solicitations for relief from the wretched appeal to my feelings, without a response as far as my means would permit. The intuitive sympathies of the finer feelings of humanity were powerfully elicited by such objects; and had I but possessed the moral courage to overcome, by self-denial, the craving desires of my sensual appetites, I had not been such a "blot" in creation. These were the anomalies that existed in my nature, entirely owing to early impressions and indulgences not corrected by proper example and education—an evidence that none of Nature's laws can be infringed with impunity, and that reason, which renders man pre-eminent, can never be misapplied without punishment.

Thus, again, after a hearty good-bye to the family household, did I again resume my old employment of "handling the ribbons," professionally so called. My route this time was over part of my old ground, sixty miles per diem, thirty miles up and down. On arriving at one end of my destination, at eleven o'clock in the morning, I had no useful object in which to employ myself till the hour arrived for resuming my duties on the coach—five o'clock in the evening; and thus were left six hours in a large fashionable town, to be spent in idleness or folly, the enervating influences of which tended still further to corrupt and debase my moral nature. Two brother "whips" arriving at the town about the same time as myself, we generally met, soon after, at a friend's, who carried on the business of a brewer and cooper, where, seated in a homely manner on some of his half-finished barrels, we were wont to discuss sundry pots of his eightpenny ale, till time reminded us of the near approach of the dinner hour, of which meal we invariably partook at a tavern kept by a former "knight of the whip;" but before reaching our restaurant, a half-way house was selected, for the purpose of swallowing a glass of gin and bitters, as a provocative to appetite, and as an assistant of the powers of digestion. This was the only meal during the twenty-four hours I could indulge in, to do which it at last became necessary to imbibe a vast quantity of stimulants, sufficient to make a moderate drinker inebriated, to artificially produce the requisite incentive. Grog and tobacco occupied our time till the arrival of the hour for my return; and as diluted spirits, or ale, formed my "refreshment" at each change of horses, on my arrival at the end of my journey I had, to every one's observation, partaken of more than a "*quantum suff.*" consistent with ebriety. I might even then have escaped worse consequences, had not the politicians of the town, awaiting with impatience the oracle of information just imported from St. Stephen's, seized me with a "will-ye nill-ye" sort of manner, and installed me leader of the night's carousing. Thus, from a too yielding disposition, I was fast progressing on the road to ruin. This became my daily routine; and well I recollect the envy with which I beheld my brother whips, with stronger constitutions and unimpaired stamina, partake of these indulgences with impunity. At last, after several months of the same debasing practice, one night, late in the autumn, within ten miles of my destination, I was seized with a fit, half-drunken, half-apoplectic, and precipitated from the coach-box to behind the horses' heels, one movement of which would, in all probability, have consigned me to eternity; but, through God's providence, the poor animals intuitively stopped, and, half-stunned, though partially sobered by the fall, I crawled from underneath the coach, and again mounted the box. Having at the time no passengers on the coach, the circumstance was never known. This had the effect, for a little time, of making me more careful; I had even firmness enough to risk the displeasure of my political friends, by immediately going home after leaving the coach. I felt, at the time, no ill effects from the fall; but I have no doubt it had something to do in producing another approach to delirium tremens, with which I was about this time visited. I was awakened one night by a number of persons, who seemed to be calling me from the road, underneath my window, to come down and join them. I awoke my wife, and asked her the meaning of it: she did not understand me. I told her that a number of persons were calling me, from outside the house, to come down and join them in their pursuits; some were going out shooting, some horse-racing—all about to proceed on some object of business or pleasure. The voices were so numerous that they appeared to proceed from untold millions. As I persisted, with every appearance of rationality, in the reality of my assertions, my wife very naturally asked me "How so many persons as I described could find room to exist in so confined a space?" I told her that that difficulty was all made easy and clear to my apprehension. The voices I heard, as voices, were all emanations from disembodied spirits; their immaterial being or essence, taking up no room, enabled them here to remain in the same locality which they inhabited in the flesh. Some voices appeared to my imagination to proceed from those who had been dead for centuries; others, who had departed at a more recent date, some of whom I had well known; all seemed to be talking about their different engagements, as in the days of their materiality; and those I had known I recognised by their voices. One said, "Leave him alone just now; he wont be with us yet; his time is not yet come." Such was the shape this fantasia took on this my second attack of "mental aberration," or rather, "mental reality."

How long I was confined at home in consequence, or whether confined at all, I now forget; but not one iota of the foregoing impressions, so indelibly are they engraven on my mind, can ever be forgotten. I continued my duties on the road for about ten months after this, nothing occurring to call for particular notice, except a day's holiday occasionally, to get rid of the effects of a debauch the preceding night, when one morning—a summer's morning—after having for a week completely saturated myself with "the accursed thing,"—so much so, that it resembled "burning lava" in my veins, and I only wonder spontaneous combustion had not taken place,—I was driving, or rather, attempting to drive, into a town, as early as eleven o'clock in the morning, in a shocking state of intoxication. I was discharged in the greatest disgrace, my character lost, my health impaired, and with no distant prospect of absolute starvation. But, blessed be God, the Father Almighty, who did not, as I deserved, in this my aggravated wickedness, utterly forsake me. A gentleman of the neighbourhood, taking compassion on my little helpless family, and hoping that these sore trials had worked in me reformation, which I, vainly trusting in my own strength, hoped as well, procured for me a subordinate Government situation in the Great Metropolis, but at so low a weekly wage, that my wife's greatest economy made the sum scarcely suffice to keep the children clothed and fed. The situation I now filled was of that nature that brought under my notice many curious incidents—*faux pas* in high life; the revelries of the disciples of Bacchus, and sports of the votaries of Dame Venus, in middle and lower life; the cunning of the sharper and the ingenuity of the thief, each driven to the practice of his art by dissipated habits or improvident waste. But as a relation of these will only give us a further insight into man's depraved nature, which my own personal history sufficiently illustrates and exemplifies, I will forbear to give it, and briefly bring to a conclusion this part of my narrative of life's past experience, by stating that, after about nine months' servitude in the above situation, disgusted with my calling, and still under the necessity, imposed on me by my previous habits of inebriation continuing, my discharge was again rendered necessary; and thus once more I became as the "offscouring of all things," shunned as a pest, and pointed at with the finger of scorn as a degraded type of human depravity.

Notwithstanding the abandonment of myself to the influence of the intoxicating cup, and the misery and desolation that I had so frequently brought on myself, my dear wife, and little ones, my love for them was most undoubted, *malgré* the assertions to the contrary of the cold, phlegmatic, and uncharitable moralists whose peculiar "temperament" allows not Bacchanalian excesses to be their besetting sin. Each individual has "a sin"—a sin which his nature and his disposition render peculiarly his own—avarice, ambition, love of frivolities and dress, pride, envy, the giving oneself up to the pleasures of the world;—all these, without the grace of God prevent, will as much render the soul unfitted for being the recipient of the spiritual and refined delights of heaven on earth, as those grosser and more sensual sins which, from their glaring nature, more conspicuously attract the notice of fellow-sinners. *Yes! my children I loved!* But when sober, and capable of reflection, I could not bear their caresses; their innocent prattle went like daggers to my heart; the tears of my much-injured and affectionate wife, when, under the most agonizing feelings, she implored me, for her sake, the children's sake, and for my soul's sake, to abandon the vice that was fast bringing her and the dear children to the workhouse and the grave, drove me to madness, and, frequently rushing out of the house, I have been ready to cast myself from one of the bridges, in the vain hope that, by ceasing to live in this world, I could produce on my mind the effect of fabled "Lethe's stream," and that, by the act, past recollections could be buried in oblivion.

At this particular time, 1837, in a large city, totally unknown, without a friend or a relative to whom I could apply for advice or employment, a very few days or weeks must have found me a claimant on "parish relief." Everything looked dark—there appeared no bright side—not one glimmering ray of hope to guide me in the way by which to procure employment to sustain my all but starving children. Ah! poor, distressed, burdened sinner, never despair! "thy Saviour liveth!"—"Christ is all in all!"—"thy God is a God of love!"—"He is a Father to the fatherless; a very present help in time of trouble." How often have I noticed the providence of a wonder-working God manifested at the time of my—and man's—greatest necessity! The more abandoned and shunned by the world, the closer is He at hand to give relief to work His way.

My wife, in canvassing for votes to procure admission for my eldest son—one of seven (*alas! since unhappily wrecked and drowned at sea*)—to St. Ann's School, Brixton, raised me up a friend in the person of the humane and much-respected secretary of that institution, who, although it was not in his power to meet our wishes regarding my son, yet used his interest in procuring me a situation in a "Company" then in course of formation, which once again, by God's blessing, placed within my reach the means of providing for my family.

This was in October, 1837, and with this "Company" I still (1853) remain. Nearly sixteen years have gone by since I entered their service, twelve years of which, though I drank a great deal, I never went the same lengths in my besetting sin; after which time the raging desire of my old lust again overcame me, bearing down all opposition, carrying me captive wheresoever it would, and in consequence of which had again been nearly the means of casting me loose on the world's cold charity, the relation of the cause and effects of which will form the sequel of this my narrative.

I would, by way of preamble to this sequel of my life, call to my and the reader's recollection, how often it has been our lot to notice the readiness with which persons convicted of gross error in moral conduct, or on matters of business, make strong efforts and resolutions of future amendment and caution, which resolves are fully acted on till the next strong temptation to err, which is given way to from the sheer necessity that the habit has imposed on us. Habit—all-powerful, prevailing habit—how gifted to reconcile all the contradictions to which our nature is prone: the miser his gold, the man of business his calling, the sensualist his pleasures,—all of which survive in the mind through habit, the physical energy of the body necessary for the accomplishment of its purposes.

On the 2nd of October, 1837, with a joyful and thankful heart, I entered on the duties of my new calling, resolutely determining, in *my own strength,* to avoid the abuse of the intoxicating cup. Ah! little did I know at that time that nothing but the totally ceasing to taste of alcoholic drink could cure excess in such a temperament as mine—I, who for the greater period of my life had lived but by stimulants, and by partaking of the least drop of this "venom of asps," lost the power over my will, and was led by the syren captive. I had, very shortly, by indulgence again in my fatal propensity, more than once nearly lost my situation, when by some means "teetotalism" was introduced to my attention; and reading shortly afterwards a notice of the intended opening of "Chelsea Temperance Hall," 1838-9, I mentioned the circumstance to my wife, who most readily embraced the proposal of attending the opening of the Hall; and it was mutually agreed that we should both sign the pledge,—a determination taken on the part of my wife as an encouragement and example to me, she being at all times an extremely abstemious woman. After hearing several stirring speeches, showing the inutility of these drinks, either for the purpose of preserving health or of imparting strength for labour, we both signed the much talked-of pledge, now upwards of fourteen years since, which pledge my dear partner has consistently and conscientiously kept, notwithstanding the importunities and the ridicule of *professing* friends. Now, what did this total abstinence from the drunkard's drink give rise to in one who, for nearly thirty years of his life, had been a confirmed inebriate? Why, in less than twelve months from the time of signing this pledge, the attendant changes from levity and carelessness, to thoughtfulness and sobriety, were the means of more fully developing the experience I had gained in my previous situations in life; and some improvement which I suggested in the working of the business of the Company in which I was employed, being appreciated and acted on, I was promoted to a situation more than equal to my expectations. My health, from the change in my habits, for the first six or seven months wonderfully improved; my spirits were of that buoyant nature, in consequence, that I could hardly restrict myself to *walking* in the streets, but felt inclined to lay aside the rules of decorum, and run. My pledge was sacredly kept for fifteen months, when, suffering from some ordinary and simple indisposition, I applied to a *medical man,** who, on learning the nature of my dietetic habits, ordered me to drink two glasses of *stout* per diem, observing at the same time, that if I continued in my plan of total abstinence from alcoholic drinks, I should soon be food for the worms. I unfortunately "listened to the voice of the charmer," vainly hoping that my past experience of the horrors of slavery to the habit of indulgence in strong drink, and the self-denial I had exerted, during the fifteen months I had kept the pledge, would have kept me from ever more becoming its victim. Vain hope! mistaken self-reliance! And I would here digress, to earnestly press on the attention of any young man whose eyes these lines may meet, should he unfortunately already have contracted a liking for the inebriating cup, which merely the love of company and good fellowship may excite, though he may as yet avoid excess, take care that use, in time, does not produce a confirmed habit, which will certainly in the end lead him to the drunkard's grave. Few men have self-command over their moral powers,—the same, after the excitement of the first glass, which they before possessed. The more that he feels himself exhilarated by the pleasing draught, or, as he expresses himself, "the more good it does him," the more he should be convinced that his temperament is the one above all others that should cease the indulgence. The foregoing advice of some of the faculty has sent thousands back, as "the dog to his vomit again, and the sow to her wallowing in the mire." Pause, ye medical professors of the present day, ere you prescribe that to your patients which is causing the ruin of millions of souls.

* This very surgeon, I have recently been informed, is dead—cut off in the prime of life, principally through his indulgence in this vice!

Thanks be to God's providence in enlightening men's minds to search after truth—thanks be to Him for the new light thrown on this question by students of physiology in the present day! We know that there are medicines, as tonics and stimulants, in the "Materia Medica," much more effectual than alcohol in any form or shape, and which, if always substituted medicinally in the place of the body and soul-destroying leaven, would tend much to bring into disrepute the dangerous practice of making these drinks a fashionable daily beverage, and thus save many a backslider. Thus, as I before observed, after owing my promotion to total abstinence, and after having strictly kept by my pledge for fifteen months, I was encouraged by this son of Esculapius' advice to again partake of the "accursed thing," the chief instrument in Satan's hands for trepanning the souls of men. Two glasses a day, as might have been expected, did but for a little time; my business taking me out of doors a great deal, the importunity of my friends—so called—when they found that I was no longer a strict abstainer, quickly overcame the reluctance I at first felt to increase the quantum. The natural result very quickly followed, and I not unfrequently found myself what is commonly called, "disguised in liquor."

This state of things continuing for some seven or eight years, alternating between abstinence and the excessive use of the beverage, I could never touch it with impunity. Total abstinence or gross inebriety was my practice. When I hear one person tell another that "he should do as he does, leave off when he has had enough," I compare such advice to one person carrying a certain weight a given distance, and another being blamed for inability to carry the same for more than half that distance. At length, after continually sipping every half hour for days, for the sake of keeping up the necessary stimulation—eating nothing—the sought-for provocative of these beverages at last ceased to be felt; they completely failed even in their intoxicating property; I called, on my way home in the evening, on a gentleman of my acquaintance, and was describing my feelings to him very rationally, as he afterwards informed me, of having the apprehension of some great calamity hanging over my head, an indescribable sensation bordering on despair, which drink no longer seemed to have power to dispel. He sympathized with me, and advised me when I got home to wash myself over with tepid water, well rub myself with a rough towel, and go immediately to bed. On reaching home, my wife was surprised to see me so sober, rational, and apparently composed. I acted on my friend's recommendation, and retired to bed, with the vain hope of composing myself to sleep. I shall never forget that night, so fraught with horror, that even now, at nearly six years' distance, my blood curdles at the thought. "Whether in the body or out of the body," as the holy apostle St. Paul describes, "I know not;" it was not natural, though it might have been magnetic, sleep. I found myself walking in a large and rather secluded street of an extensive city, in the dusk of the evening, when suddenly, a loud cry—not in words, it seemed to me, but a cry that shook all nature, and that all creation felt—"The Son of Man cometh!" And immediately, on my looking up, I beheld an innumerable host of angels, which no man could number; they appeared to fly in a sort of wedge-like form, the leading angel, larger than the others, having a trumpet at its mouth; those that followed gradually grew less in size and greater in number, assuming a shape something like what we have seen represented of the cherubims. They flew "steadily swift," as an eagle's flight; the smaller angels darting down and gathering up the people in the street, like the eddying whirlwind of a summer's day gathers up the sticks and straws and dust. We were thus caught up in knots of eleven or twelve persons, of all ages and sizes indiscriminately; one or two old men, with girls and children, were in the lot I was whirled up with. Each individual, judging by my own experience, had his doom fixed. I was immediately changed from the form (though possessing the same feelings) of a human being, into that of a globe,* a ball, or, indeed, as it seemed to me, in reality and shape, a world of darkness—darkness the most intense—fated to revolve on my own axis, in an orbit, round an amassing centre of gravity, a fixed sphere of darkness. The Spirit immediately told me, or rather the fact emanated from the Spirit, that this state was that of the existence in "everlasting blackness and darkness of despair,"—that as the sun, in the system of the visible universe, was the great centre of attraction, giving out warmth, and light, and life; so, on the contrary, this dark central globe of attraction was hell, Satan's home, from whence proceeded misery, despair, and woe, to endure for endless ages. It seemed that I revolved on my axis exactly on the same principle as that of the earth—continually turning round. I was always in the greatest agony; full to repletion, never empty, though the contents of my stomach were continually leaving me; under the most intense sensation of sickening agony, yet never lessening the overpowering sense of fulness. This was to be my punishment for ever and for ever, for having so grossly abused the natural appetites which God had given me for the sustenance of my body. I had so revolved about three times, though it seemed ages, under the torture of mind and agony of body, when I returned to this world; in other words, I returned into and

* Every human creature is a world *to himself, in himself;* his sins, his virtues, thoughts, and feelings, all emanate from the living principle within, by which his future state will be governed.

quickened the body. How long this magnetic trance-like state continued, I know not; but of so appalling a nature was the impression produced on my mind by the settled conviction of the reality of the tortures endured, and was, I felt persuaded, to be reimposed on my final entrance in my spiritual state to the unseen world, on the dissolution of my body, that I could not bear its contemplation, but endeavoured all in my power to anticipate my fate by attempting to dash my brains out against the wall, as no other means presented themselves to my notice with which to accomplish my purpose. This continued a day or two, every portable piece of furniture being removed from the room. Continuing still to agonize on these reflections, my wife earnestly entreated me to appeal to that Being, through the medium of a Saviour, whose ears are always open to the cries of the distressed. This induced me to turn my thoughts to that source of help, feeling myself to be a condemned, lost soul. The "great enemy of mankind," for a long time, urged that my sins were of too dark a dye to find forgiveness; that I had sinned against light and against truth, and had crucified the Son of God afresh, and nothing was left but a fearful looking forward to a fiery indignation from judgment to come. But to these and other suggestions of Satan, I seemed to have on the other side of me, as if from the breathings of angelic natures, sentences of Scripture full of hope and love, as an answer to these condemnatory charges, urged by the "arch-enemy of man."

These delusions—no, these realities, fearful realities!—assumed a different shape from those I have before related, inasmuch as then, voices and utterance of words were distinctly heard. Now, on the contrary, I felt intuitive spiritual emanations, that seemed to proceed from two distinct intelligences within,—one leading to hope, the other driving to despair. As these urged themselves on my mind, I seemed under an irresistible power, obliged to give utterance to them; when a word failed me to complete the passage—for they were all texts from Scripture—my wife would assist me by supplying the necessary word, for every fresh hope seemed well grounded only as the enemy's accusations were answered from the Book of Life. For these I would bless, thank her, and call her "Eve." She begged me not to call her "Eve;" but I looked hard at her, and saw clearly enough that she represented in herself the first mother of mankind,—she appeared pure in her nature as before the fall,—and the words and sentences to which she directed my attention seemed to me, by the Spirit's suggestion, to represent links in that perfect chain of the Word of God that was to bind the "Deceiver" throughout eternity. This importuning God, through the Saviour as a Mediator, went on for some nights and days with but little intermission, the absolute reality of past horrors still keeping possession of my mind, till at last my medical attendant had great doubts of my recovery, and asked "how it was, at intervals, I was always wanting the Bible to read, when before my illness, as he had been told, I had shown no particular predilection in its favour?" I told him what was then the fact,—that in times past I did not understand its contents, but that now I seemed to read and understand every line, both in its natural and spiritual sense. I had evidently, to every one's perception, undergone some mental change; some mental magnetic spell was on me; every incident in my past life was palpably before me, engraven on my mind as on a book; whole sentences, nay, nearly whole chapters of God's word I could quote from memory, though I had not read them for years before. I made my son write from my dictation what memory spontaneously brought forth as applicable to my spiritual state. The sense no longer seemed ambiguous; every sentence seemed to contain a world of meaning. I no longer wondered, as heretofore, at the punishment mentioned in the Apocalypse for taking away one word from the Book of Life, that God should take away his part out of that Book of Life; and I moreover now see the truth, and its applicability to the sinner, of what is said in the Gospel of St. John, that "in the beginning was the Word, and the Word was with God, and the Word was God." Appealing to this God through my Saviour as a Mediator, and answering in this Word my "persecutor," God in me gained the victory. At one particular time, whilst engaged in my intense supplications, I suddenly saw the whole of my children, seven in number, and wife, standing at the foot of the bed, surrounded by a halo of soft and heavenly light; I felt under the conviction that the meaning of this vision was, that I saw them represented in this state as saved souls. Glowing with surprise and pleasure at this sight, I looked intently again, and exclaimed, "What! all?" The Spirit answered me, "Aye, all!" I had scarcely realized the joy it gave me, when my adversary whispered, "You do not see yourself there!" My joy vanished, my fears returned; I again, like Jacob with the angel, renewed my importunities, and wrestled with God, and exclaimed, "I will not let Thee go except Thou bless me." At length, in the midst of my agony, I saw above a scarcely perceptible ray of light,—that ray, that little twinkling ray, was like life from the grave. In fervour my prayers increased. The light grew stronger; I felt more composed than I had for days; my fears allayed, I slept a blessed sleep (I had not closed my eyes for thirteen days); for, on awakening, I felt that indescribable "peace of mind which passeth all understanding." Whilst blessed with this feeling, my wife came into the room: I sat up in bed, and told her that I was perfectly happy. I said (it seems now to me presumption), "I am a saved soul,—my sins are forgiven me." On something being mentioned about the probable loss of the situation I filled, about which I had previously been so anxious, I said, "I am in God's hands, I fear nothing: God has said, 'Seek ye first the kingdom of God and His righteousness, and all these things shall be added unto you.'" My fears on this head entirely disappeared.

From this time I gained strength rapidly—so much so, that my medical attendant was astounded to see me so soon, after this three weeks' illness, walking with as elastic a step as though I had never been ill. When convalescence returned, still in the enjoyment of the indescribably happy state of mind before spoken of, I felt an irrepressible desire to again join the ranks of the total abstainers, and accordingly took the first opportunity which a meeting presented of again signing the abstinence pledge, which was accompanied by earnest prayers that I might not again swerve from it; and during the nine months I observed this, I was the happiest of mortals. Nature in me seemed exempted from the ills of mortality; everything I looked on—the works of nature or of art—seemed to call forth thanksgivings to the great Creator. Looking up at the starry firmament at night, and gazing on its splendours of revolving planets, of fixed stars, suns of other systems, and the great and wondrous whole,—so absorbed my feelings were, and so filled my soul with awe, wonder, and love, that I had some difficulty in restraining myself from kneeling down, in the face of man, to pour forth my gratitude to the great Maker of all for his love in enabling me to be a partaker in joys so great.

"Ah!" I think I hear my reader exclaim, "why this pause? What! again, after being brought to that happy state, that realization of heaven upon earth, that millennium state of blessedness which will be enjoyed by His people when 'His will is done on earth as it is in heaven,' which the great movements of the present day seem fast making way for,—after being a participator in all these unhoped-for blessings, hast thou again done despite to the Spirit of Grace—sinned against light and truth, and trampled under foot and crucified the Son of God afresh?" Reader, I have!—I had committed the unforgiven sin! But God is love.

I said about nine months I enjoyed this state of happiness, when my Adamite nature again led me into sin; neglecting the means of grace, I became careless, and fell from this height of spiritual fervour, which seemed, when gone, to be lost for ever. Sin is ever progressive; there is no pausing in the downward course. Satan once again got the victory, and made use of his subtle and specious arts by stifling convictions, to lead me again into that abyss from which I had before so frequently, by God's special grace, been extricated. I again partook of the drunkard's cup; and so bound over, body and soul, was I again to its servitude, that I abjured for ever, as a monstrous error, the total abstinence doctrine, and avowed that I would never again become converted by its sophistry and fallacies. I continued under this delusion four years, occasionally taking less, as my feelings of coming ill-health at times admonished me. It was about this time alcoholic drinks seemed to produce on me a different physical action; and instead of getting stupidly and insensibly unconscious, my intellect and apprehension seemed, under its influence, astonishingly to expand. I could carry on my argument, on any subject with which I was at all acquainted, with such method and vigour, that neither I nor any of my companions considered me to be drunk. I essayed to take my departure, when, much to my surprise, I seemed to have lost the locomotive power, and as a policeman observed, when I told him that I was drunk, that if so, it was my legs and not my head, for that I talked too reasonably for a drunken man. In this manner I continued sipping every half-hour, every day, for several days, as I could not eat, with an idea of stimulating myself to that exertion necessary for me to fulfil the duties of my calling. The result can be foreseen—again delirium tremens, or a species of it, attacked me, and now in a more fearful manner than ever. This was about the end of July, 1850.

It seems to me, in looking back to this period of my experience, that Satan gains more power over the soul of the inebriate each time that he falls into this state of syncope. I believe it is a state in which God leaves the soul for a time—in some cases, for eternity—entirely abandoned to Satan's power. Again his influence was constantly exerted to cause me to commit suicide; and nothing but my soul being occasionally urged to prayer by ministering spirits, prevented the accomplishment of his purpose. In this condition I still continued mechanically to fulfil the duties of my station, but, no doubt, in so strange a manner as to attract the notice of all with whom I had any business to transact; and yet I possessed sufficient tact to make my singularities attributed to any other than the right cause. Again I had my sleepless nights and restless days, harassed with the most fearful forebodings, and threatened by the denunciations of the "wicked one." All the sins of my past life were brought individually and collectively with fearful distinctness to my view; each separate sin seemed mysteriously to bear its part to work out that retributive justice with which I was then being punished. The veil seemed lifted that concealed the torments of eternity. I then felt that it was no corporeal punishment in the next world that awaits our sins, though typified in this world by a natural element—fire—the sufferings inflicted by which our materiality can realize. It is the mind, the conscience, the soul, that immaterial part of us, that is to realize the torture typified by burning brimstone and fire—a fit emblem of its reality. Again the demon made use of the same weapons he had heretofore used, but with far greater power and success; I seemed entirely to despair under his accusations and threatenings, verified as they were by the "Word." I was, after great agony, enabled to answer him by some word of truth in its proper interpretation, suggested to my mind by some heavenly antagonistic spirit; he was no sooner driven away by one reply, so suggested, than he brought another more fearfully condemnatory to my soul's views—so I continued to be tormented until I found myself, as in my former experience, out of the body in a liquid lake of molten fire. In my feelings on this occasion there is an anomaly. I seemed to realize the torment inflicted by the liquid lake but for a moment, and yet I seemed to be there for ages. I was irresistibly impelled to call out, in the spirit, so loud, that in that fearful place it seemed to reach immensity, the following words—"How long, Lord? how long?" These words I was commanded by the Spirit to call three times; and so loud did I call, that it seemed to shake the foundations of earth and hell. On coming to myself, or on returning into the body, methought it necessary, to carry out God's purpose, that the same cry should be made in the flesh, which I essayed to do, but was, in my attempt to give utterance to the words, stopped by my wife placing her hands before my mouth, and calling down my eldest son to assist her. I looked on her, and said, "What, Eve!" (for she still appeared to me, in the state I was then in, to again represent our first parent, "Eve")*—"You suppress the word of life? you that formerly gave to me the word"—alluding to the experience on a like occasion some years back. I looked hard into her eyes to see if it was indeed "Eve." Yes, she was the first mother; but there was an expression in her features different from that heavenly look she had when I first identified her as "Eve." On asking her the meaning of the change, the Spirit answered me—"You saw her then before the fall, in her original purity; you see her now after the transgression, contaminated by sin; hence the wicked part of her nature induces her to assist the wicked one in suppressing the Word of Life." Thus encouraged by the Spirit, I struggled, and eventually succeeded in giving, in a loud cry, utterance, with much opposition, to the predestined words, "How long, Lord?—how long?" three times; which

* Long since the above was originally written, I accidentally came across the derivation of the word "Eve," from its original meaning. It is, "*Life-giver.*"

seemed, as before, to reach to the confines of the earth. On my last call, I was answered by the words—"When death is swallowed up in victory;" and the Spirit moreover told me to answer all my spiritual tormentors with those words, and "to rest there." He then added, "You are sealed." I supplicated to be informed whether for "glory" or "perdition," but could get no further answer. The only hope I had was from his before telling me "to *rest* there."

*The Conclusion.—Reflections.*—As I lay on my bed gradually recovering from the effects of the foregoing excesses, my thoughts became insensibly engaged in analyzing the act of the Saviour in turning water into wine at the marriage feast at Cana at Galilee, and of His administering wine to His disciples on His last partaking of the Passover, on His instituting the Eucharist. Was it possible, I asked myself, that our blessed Lord would set an example of partaking of that beverage, the use and abuse of which would hereafter become a curse and reproach to us as a nation calling itself after His name,—more especially as a great majority of physiologists have declared the use of it to be not only unnecessary to a human being in a state of health, but to act injuriously on the finer tissues of the body, and otherwise disarrange the more susceptible part of man's nervous system,—it being a well-known fact that some constitutions are of that delicate construction, that not the least portion of alcoholic drink can be partaken with impunity, without giving a false colouring to objects, by which such person's self-denying power is lost, and thus causing them to err? Whilst these thoughts were revolving in my mind, I again seemed to have the same powerful agency telling me "to search!"—"search!"—"search!" till I was impelled, as it were, to get out of bed and get the Bible to satisfy my doubts. Under the same irresistible tuition, I sought for that which preceded the Eucharistic rite of the Passover, the formalities of which institution I found in the 12th chapter of Exodus, verse 15—"Seven days shall ye eat unleavened bread; even the first day ye shall put away leaven out of your houses; for whosoever eateth leavened bread from the first day until the seventh day, that soul shall be cut off from Israel." Verse 19—"Seven days shall there be no leaven found in your houses." Exodus, chap. xxxiv. ver. 25—"Thou shalt not offer the blood of my sacrifice with leaven." Now, had alcoholic wine been used on that occasion, it must have been leavened, fermented, and undergone the first process to decay, which would have been a very inapt emblem of "the Blood of the New Testament,"—that blood that flowed from that body of our Blessed Lord, that had in it no element of decay. The human nature, derived from his mother

Mary, had been so purified and made perfect by suffering, and overcoming temptation, as fitted that body for becoming the residence of His divinity, and by the process purged it of all elements of dissolution. But it is useless considering further, or arguing a point which is not disputed by those who have made themselves acquainted with the late manners and customs of the Jews. There were two sorts of wine used in those days—one fermented, leavened, or alcoholic; the other, and most commonly used, as being most congenial to their tastes and the habit which their climate produced, unfermented, of the expressed juice of the grape, before leaven, that "type of sin," had been applied to it. The wine used at the Passover of the Jews of the present day is, I am given to understand, of a somewhat similar nature—is unalcoholic. Believing this fact, many eminent divines of the Christian Church (nonconforming) make use of the unchanged element when acting on this command of their Master, to "do this in remembrance of me." Somewhat exhausted by the mental exertions used in the investigation of the above subject, I lay back on my pillow, with the hope of composing myself; but a short time elapsed before I was again "acted on by the Spirit" (I use this phraseology, as the impulse given seemed of that uncontrollable nature as to seem supernatural) with the same "Search!"—"search!" I seemed to be for a time satisfied, but still more urgently did the Spirit tell me to "Search!"—"search!" I again got the Bible, and found out our Lord's institution of the Eucharist, which I came across at the 14th chapter of St. Mark's Gospel, 12th and following verses—"And the first day of unleavened bread, when they killed the Passover, His disciples said unto Him, Where wilt thou that we go and prepare that thou mayst eat the Passover? And He sendeth forth two of His disciples, and saith unto them, Go ye into the city, and there shall meet you a man bearing a pitcher of water: follow him." I read so far undisturbed; but on coming to the passage, which I must needs repeat, it had such an effect on me,—"and there shall meet you a man bearing a pitcher of water: follow him,"—I became so agitated that I nearly fainted. On recovering, I was impressed with the presentiment that some great theological fact was about to be given to the world, in connexion with this ordinance, which had never been thought of before, which was still further confirmed by the "Invisible Presence" most urgently reiterating, "Read on!"—"Read on!" I read on till I came to the 22nd verse of the same chapter (14th)—"And as they did eat, Jesus took bread, and blessed, and brake it, and gave to them, and said, Take, eat: this is my body." 23rd verse—"And He took the cup, and when He had given thanks, He gave it to them: and they all drank of it." 24th—"And He said unto them, This is my blood of the

New Testament, which is shed for many." I at this part seemed to be interrupted, and was asked—" What is my blood of the New Testament ?" I paused to reflect. The question (similar) was repeated, as in another version you find it—" What is the New Testament in my blood ?" I mentally answered, " the contents of the cup,—the wine." The Spirit replied—" How do you know it was wine ? Turn to the 19th chapter of the Gospel of St. John, and the 34th verse;" and I read—" But one of the soldiers with a spear pierced his side, and forthwith came thereout blood and water." I exclaimed—" Blood and water !" " Blood and water !" said the Spirit. Thus is verified what is stated by St. John—" This is He that came by water and blood, even Jesus Christ; not by water only, but by water and blood. And it is the Spirit that beareth witness, because the Spirit is truth." Water is the type of truth. " This," said the Spirit, " is the New Testament in His blood; the water that flowed from His side. The blood showed and typified His human nature, derived from His mother Mary, according to the flesh; the water His divine. When He had perfected that humanity by overcoming temptation, and finished the work the Father gave Him to do, it was then that He bowed His head, and said, ' It is finished.' The blood of His perfected Humanity, poured out as the grand atonement by which His people received remission of their sins; the water, by the partaking of which in faith we are sanctified emblematically, and fitted by God's grace to become recipients of heavenly joys." After so far telling me, I said, on reading further—" Verily I say unto you, I will drink no more of the fruit of the vine until that day I drink it new in the kingdom of God." He replied—" ' I am the vine, ye are the branches;' read the Lord's Prayer,"—which I repeated with much reverence—" Our Father, which art in heaven, hallowed be thy name; thy kingdom come; thy will be done on earth, as it is in heaven." He stopped me to say—" Thou wast not taught to pray in the Saviour's words, for what will never be. His kingdom will come, when His will is done on earth, as it is in heaven; then will He drink the wine, new and unfermented, in His Father's kingdom on earth, when, with virtue and goodness, universal temperance shall prevail, and the Spirit of Evil, typified by alcohol, go back and reside with the Spirit who gave it." So impressed was I, at the time, with the truth of the above revelation (not having partaken of any sustenance for some time, having no desire), I immediately rose in my bed, rang the bell, and ordered a cup of water to be brought me, on receiving which I knelt down with the greatest awe and reverence, under the impression of the omniscience and omnipresence of the Deity, and enthusiastically, with much fervour, invoked God's blessing on the element which I then verily believed to typify His holy Spirit, "the New Testament in His blood."

I was then referred, as a climax to the whole, to the 44th chapter of Ezekiel, 21st verse, where I read—"Neither shall any priest drink wine when they enter into the inner court." 23rd verse—"And they shall teach my people the difference between the holy and profane, and cause them to discern between the unclean and the clean." The Spirit then left me, telling me, "I was sealed." This took place, dating from this 1st of November, 1853, nearly four years since, from which time, by God's mercy, I have not touched the unholy thing. I feel assured that when the time will arrive when "His kingdom is come, and His will is done on earth as it is in heaven," its use will be banished the earth. John the Baptist came—a water drinker—heralding in the "first coming of Christ;" so I believe this present total abstinence movement from alcoholic drinks to be a faint ray of that light which will ultimately prevail to herald in "His second coming."

---

